# Activity or connectivity? A randomized controlled feasibility study evaluating neurofeedback training in Huntington’s disease

**DOI:** 10.1093/braincomms/fcaa049

**Published:** 2020-04-23

**Authors:** Marina Papoutsi, Joerg Magerkurth, Oliver Josephs, Sophia E Pépés, Temi Ibitoye, Ralf Reilmann, Nigel Hunt, Edwin Payne, Nikolaus Weiskopf, Douglas Langbehn, Geraint Rees, Sarah J Tabrizi

**Affiliations:** 1 UCL Huntington’s Disease Centre, Queen Square Institute of Neurology, University College London, London WC1B 5EH, UK; 2 Birkbeck-UCL Centre for Neuroimaging, University College London, London WC1H 0AP, UK; 3 Wellcome Centre for Human Neuroimaging, Queen Square Institute of Neurology, University College London, London WC1N 3AR, UK; 4 University of Oxford, Harris Manchester College, Oxford OX1 3TD, UK; 5 George Huntington Institute, 48149 Münster, Germany; 6 Department of Radiology, University of Muenster, 48149 Münster, Germany; 7 Section for Neurodegeneration and Hertie Institute for Clinical Brain Research, University of Tuebingen, 72076 Tübingen, Germany; 8 Eastman Dental Institute, University College London, London WC1X 8LD, UK; 9 Max Planck Institute for Human Cognitive and Brain Sciences, D-04103 Leipzig, Germany; 10 Carver College of Medicine, University of Iowa, Iowa City, IA 52242, USA; 11 Institute of Cognitive Neuroscience, University College London, London WC1N 3AZ, UK; 12 UK Dementia Research Institute at University College London, London WC1E 6BT, UK

**Keywords:** neurofeedback training, neuroplasticity, Huntington’s disease, real-time fMRI

## Abstract

Non-invasive methods, such as neurofeedback training, could support cognitive symptom management in Huntington’s disease by targeting brain regions whose function is impaired. The aim of our single-blind, sham-controlled study was to collect rigorous evidence regarding the feasibility of neurofeedback training in Huntington’s disease by examining two different methods, activity and connectivity real-time functional MRI neurofeedback training. Thirty-two Huntington’s disease gene-carriers completed 16 runs of neurofeedback training, using an optimized real-time functional MRI protocol. Participants were randomized into four groups, two treatment groups, one receiving neurofeedback derived from the activity of the supplementary motor area, and another receiving neurofeedback based on the correlation of supplementary motor area and left striatum activity (connectivity neurofeedback training), and two sham control groups, matched to each of the treatment groups. We examined differences between the groups during neurofeedback training sessions and after training at follow-up sessions. Transfer of training was measured by measuring the participants’ ability to upregulate neurofeedback training target levels without feedback (near transfer), as well as by examining change in objective, a priori defined, behavioural measures of cognitive and psychomotor function (far transfer) before and at 2 months after training. We found that the treatment group had significantly higher neurofeedback training target levels during the training sessions compared to the control group. However, we did not find robust evidence of better transfer in the treatment group compared to controls, or a difference between the two neurofeedback training methods. We also did not find evidence in support of a relationship between change in cognitive and psychomotor function and learning success. We conclude that although there is evidence that neurofeedback training can be used to guide participants to regulate the activity and connectivity of specific regions in the brain, evidence regarding transfer of learning and clinical benefit was not robust.

## Teaser

We evaluated feasibility of real-time functional magnetic resonance imaging neurofeedback training in Huntington’s disease. The treatment group learned to regulate the neurofeedback target, activity or connectivity, compared to sham neurofeedback. However, evidence of transfer was weak. The treatment group did not self-regulate the target without neurofeedback better than controls; there was no improvement in behaviour.

## Graphical Abstract

**Graphical Abstract:**
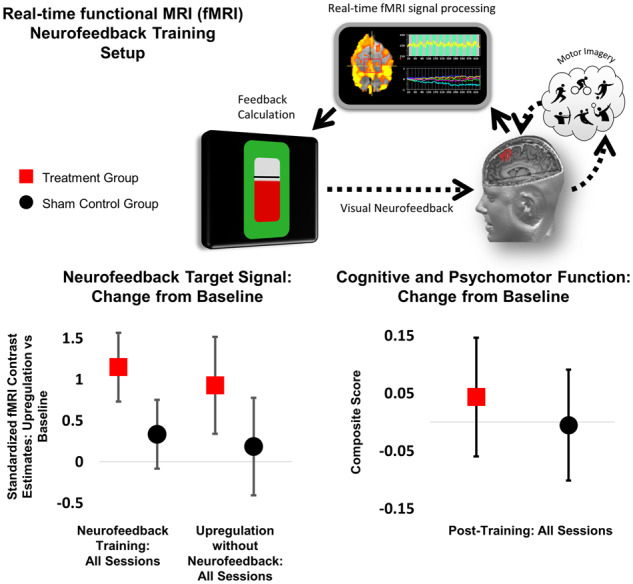

## Introduction

Neurofeedback training (NFT) is a non-invasive intervention used to train participants in a closed-loop design to regulate their own brain activity (Sitaram *et al.*, 2017). The underlying principle is that by regulating different aspects of their brain activity, e.g. regional activation or inter-regional connectivity, participants would implicitly regulate associated cognitive function. Huntington’s disease is a genetic neurodegenerative condition characterized by progressive motor, psychiatric and cognitive impairment, as well as early striatal atrophy, cortical and cortico-striatal connectivity loss (Tabrizi *et al.*, 2011; Poudel *et al.*, 2014; McColgan *et al.*, 2015; Novak *et al.*, 2015). There are currently no treatments for cognitive impairment in Huntington’s disease and the effect of disease-modifying therapies, such as antisense-oligonucleotide approaches (Tabrizi *et al.*, 2019*b*), on cognitive function is, at present, unknown. Our motivation for testing NFT, is that it, if successful, it could be used as an adjunct treatment to invasive, disease-modifying therapies (Linden and Turner, 2016; Tabrizi *et al.*, 2019*a*). However, there are several challenges in designing effective NFT trials and testing their efficacy, including the choice of an appropriate NFT target for the specified clinical population.

Because striatal atrophy and cortico-striatal connectivity loss appear early on in Huntington’s disease and correlate with cognitive and psychomotor impairment (Tabrizi *et al.*, 2009, 2011; Poudel *et al.*, 2014; McColgan *et al.*, 2015; Novak *et al.*, 2015), striatal activity and cortico-striatal connectivity would be the obvious targets for NFT. NFT could therefore be used to ‘boost’ the activity or connectivity of the striatum in Huntington’s disease gene-carriers at pre-symptomatic or early stages of the disease, i.e. while levels of atrophy are still low. In a recent proof-of-concept study, we used the supplementary motor area (SMA) as a target for real-time functional MRI (fMRI) NFT in Huntington’s disease patients (Papoutsi *et al.*, 2018). We selected BOLD fMRI signal from the SMA because it can be reliably measured in real time (Subramanian *et al.*, 2011, 2016), and its function and connectivity to the striatum is disrupted by Huntington’s disease (Klöppel *et al.*, 2009). Previous studies have also shown that NFT-induced changes are not just localized to the target region, but extend to a wider network of regions (Horovitz *et al.*, 2010; Ruiz *et al.*, 2013; Emmert *et al.*, 2016), suggesting that a proxy region would be appropriate. We found that Huntington’s disease patients can be trained to increase the level of SMA activity and that improvement in cognitive and psychomotor behaviour after training related to increases in activity of the left Putamen and SMA—left Putamen connectivity during training. This suggested that SMA-striatum connectivity could be a more appropriate NFT target than SMA activity in Huntington’s disease.

The aim of the current study is to compare the two NFT approaches, SMA activity and SMA-striatum connectivity, and to collect rigorous evidence on the feasibility of the method in Huntington’s disease. We used BOLD fMRI signal change from the SMA as the target for activity NFT and correlation between the signal from the SMA and left striatum during upregulation as the target for connectivity NFT (Megumi *et al.*, 2015; Yamashita *et al.*, 2017). In addition, we used a single-blind, randomized, sham-controlled, parallel design and employed an optimized real-time fMRI processing pipeline using a prospective motion correction system (PMCS; Zaitsev *et al.*, 2006; Todd *et al.*, 2015) for real-time head motion correction and real-time physiological noise filtering (Misaki *et al.*, 2015) to ensure that we obtain high-quality evidence. Finally, to ensure participant blinding and control for experimental exposure and motivation, participants that were randomized to the control group were yoked to a participant in the treatment group, and received feedback based on the NFT target levels of their yoked participant from the treatment group, rather than their own (Thibault *et al.*, 2016; Sorger *et al.*, 2019). This setup enables us to collect high-quality evidence regarding the use of real-time fMRI NFT for the treatment of cognitive impairment in Huntington’s disease.

## Materials and methods

### Participants

Thirty-four adults with HTT gene CAG expansion greater than 40 were recruited to the study between February 2016 and September 2017. One participant withdrew from the study due to claustrophobia at the first NFT visit. The participant had been randomized to the activity NFT treatment group and was replaced. Another participant was excluded, because a large number of trials were contaminated with motion-related artefacts. This participant was randomized to the connectivity treatment group and was replaced. Details on the remaining 32 participants who were included in the analyses are shown in Table 1. There were no statistically significant differences between the treatment and control groups for the two types of NFT (using a non-parametric Mann–Whitney test all *P* > 0.2). All participants provided written informed consent according to the Declaration of Helsinki and the study was approved by the Queen Square Research Ethics Committee (05Q051274; ISRCTN ID: ISRCTN35734205). All testing took place in testing rooms at the Queen Square Institute of Neurology and MRI scanning took place at the Wellcome Centre for Human Neuroimaging (WCHN).

**Table 1:** Demographic information

	Activity NFT		Connectivity NFT	
	Treatment group	Control group	Treatment group	Control group
Number of participants	8	8	8	8
Gender	6F, 2M	6F, 2M	6F, 2M	5F, 3M
Handedness	7RH, 1LH	7RH, 1LH	7RH, 1LH	8RH, 0LH
Age, mean (SD)	46.4 (11.3)	50 (12.3)	52.3 (11.9)	50.1 (10.3)
CAG repeat length, median (SD)	43 (3.7)	42.5 (2.1)	43 (2.5)	43.5 (1.4)
CAP score, mean (SD)	92.7 (14.2)	97.6 (11.7)	105.6 (23)	101.9 (18.3)
UHDRS				
TMS, mean (SD)	8 (12.7)	8.5 (4.3)	9 (10.1)	11.5 (14.1)
TFC, mean (SD)	11.6 (1.5)	12.5 (1.1)	12.5 (0.5)	11.6 (1.9)
MoCA, mean (SD)	26.1 (4.2)	27.6 (1.1)	25.4 (3.3)	25.5 (3.0)
HADS				
Anxiety, mean (SD)	4.0 (2.3)	3.5 (3.9)	4.3 (3.3)	4.6 (4.9)
Depression, mean (SD)	1.8 (0.7)	1.9 (1.9)	3.6 (4.8)	3.6 (3.0)
Composite Score, mean (SD)	−0.52 (0.75)	−0.21 (0.37)	−0.82 (0.67)	−0.95 (1.33)

CAP = normalized CAG-Age Product Score; HADS = Hamilton Anxiety and Depression Score; MoCA = Montreal Cognitive Assessment; NFT = Neurofeedback training; TFC = Total Functional Capacity; TMS = Total Motor Score.

Information regarding sample size calculations prior to the start of the study are provided in the Supplementary materials. Briefly, the present study was powered in order to be able to detect, previously reported, very large differences (Cohen’s *d* effect size = 1.65 and 1.60; see Supplementary materials) between treatment and sham NFT control groups in near transfer. As this was a feasibility study, we chose to power on near transfer and not far transfer effects. Although near transfer effects are not clinically relevant, they do allow us to test NFT learning transfer and as such can serve as a suitable endpoint for this feasibility study. If the findings from this study are promising, then the effect sizes estimated from this study could be used to power a future randomized controlled trial focusing on efficacy.

### Study structure

**Figure 1:**
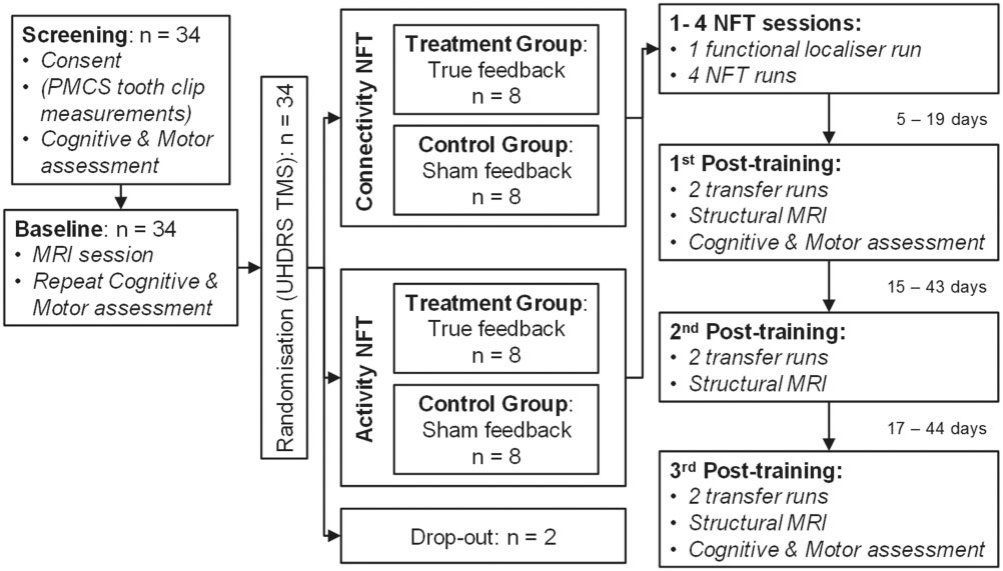
**Diagram of study structure.** NFT = Neurofeedback training; PMCS = Prospective motion correction system; TMS = Total Motor Score; UHDRS = Unified Huntington’s disease Rating Scale.

As part of the study, participants completed one screening, one baseline, four neurofeedback training and three follow-up sessions. The first follow-up was within 2 weeks from the last training visit, the second between 4 and 6 weeks and the third between 8 and 10 weeks (also see Supplementary Table 2). A diagram of the study design is shown in Fig. 1. A PMCS was used to correct head motion during scan acquisition (Zaitsev *et al.*, 2006; Todd *et al.*, 2015). Using the PMCS allowed us to recruit participants with moderate chorea without substantial data loss due to head motion. Details of the PMCS are provided in the Supplementary materials. Participants who consented to the use of the PMCS had teeth impressions acquired during the screening visit by a qualified orthodontist (N.H.). The teeth impressions were used to create a small retainer on which the optical marker for the PMCS was mounted. During the screening session, participants completed the Montreal Cognitive Assessment test (Nasreddine *et al.*, 2005) and a number of cognitive and psychomotor tasks. The purpose of the testing on the screening visit was to familiarize the participants with the tests and minimize practice effects during follow-up. The same measurements were repeated during the baseline and follow-up sessions.

To assess change in cognitive and psychomotor function following NFT, we calculated a composite score using the same procedure and measures as in our previous study (Papoutsi *et al.*, 2018). In summary, these measures were selected a priori based on previous work showing that they are sensitive to disease progression (Tabrizi *et al.*, 2009, 2011, 2012, 2013; Poudel *et al.*, 2014; McColgan *et al.*, 2015; Novak *et al.*, 2015), they were converted to z-scores and summed to create the composite score. The measurements included were: number correct for Stroop Word Reading, number correct for Symbol Digit Modalities Test (SDMT), annulus length for Indirect Circle Tracing (log transformed), number correct for negative Emotion Recognition, inter-tap interval and standard deviation of inter-onset interval (log transformed) during speeded tapping with the non-dominant index finger, and standard deviation of mid-tap interval deviation from target rhythm (log transformed) for paced tapping with the non-dominant index finger at 1.8 Hz.

The baseline and follow-up sessions included: (i) repetition of the cognitive and psychomotor testing (only on the first and third follow-up), (ii) structural MRI measurements and (iii) two fMRI runs assessing the participant’s ability to upregulate the target NFT measures without neurofeedback. The fMRI runs consisted of five upregulation blocks (30 s each), six rest blocks (30 s each) and five response blocks (18 s each; Supplementary Fig. 2B). Similar to our previous study (Papoutsi *et al.*, 2018), we used a simple attention task during the rest blocks, whereby participants monitored changes in the luminance of a white bar. After the baseline session participants were randomized to one of four groups: activity NFT treatment and control groups, and connectivity NFT treatment and control groups. Randomization was based on the Unified Huntington’s Disease Rating Scale (Huntington Study Group, 1996) (UHDRS) Total Motor Score (TMS). More details regarding the randomization procedure are provided in the Supplementary materials.

NFT sessions started with a fist-clenching run used to select the target regions of interest (ROIs). Participants were instructed to clench their left fist during the active blocks (10 blocks, 20.4 s duration) and rest during the rest blocks (11 blocks lasting 20.4 s each; Supplementary Fig. 2A). Using Turbo-Brain Voyager (Brain Innovation, The Netherlands) the fMRI run was analysed in real time and the resulting statistical map was used to define the ROIs for the subsequent NFT runs. Participants completed four NFT sessions on different days and each session included four NFT runs (two participants completed three runs on one of the NFT sessions, because of fatigue). The activity NFT runs were structured as blocks of rest (30 s block duration), response (18 s) and upregulation blocks (30 s) repeated five times, with the addition of a rest block at the end to ensure that the Hemodynamic Response Function (HRF) for the last upregulation block is not cut-off (Supplementary Fig. 2C). The rest and response blocks were identical to those of the near transfer runs and included a simple attention task as a control condition similar to our previous study (Papoutsi *et al.*, 2018). During the rest blocks participants monitored changes in the luminance of the white bar, if the white bar flickered to grey (for 1 s in three out six blocks), they would clench their left fist once, when the question mark appeared on screen (response block). During the upregulation blocks feedback was presented continuously in the form of a red bar, similar to our previous pilot study (Papoutsi *et al.*, 2018) which showed promising results. In the treatment group the height of the red bar represented the per cent signal change at a given point during the upregulation block versus the mean activation during the preceding rest block. Once the upregulation blocks started, there was an average delay of 2 s until the red bar appeared and then it was updated every 1.2 s.

In the connectivity NFT runs feedback was presented intermittently at the end of the upregulation blocks in the form of a red bar. We selected intermittent instead of continuous feedback for the connectivity NFT because it provided reliable estimates of the ROI correlation by integrating over 30 s consistent with previous research (Zilverstand *et al.*, 2014; Megumi *et al.*, 2015; Yamashita *et al.*, 2017). The connectivity NFT runs were structured as blocks of rest (30 s block duration), upregulation (30 s), feedback (3 s) and rest (15 s to allow the HRF to return to baseline) repeated five times (Supplementary Fig. 2D). Despite the differences in the runs structure, the number and duration of upregulation blocks (5 blocks, 30 s each), as well as the interval between upregulation blocks (48 s) was the same for both the activity and connectivity NFT runs. In the treatment group, the height of the red bar was calculated using the Pearson’s correlation coefficient between the SMA and left striatum ROI time-series during the upregulation blocks only (Megumi *et al.*, 2015). In both cases (activity and connectivity NFT), the feedback provided to the sham control groups was calculated using data from a yoked participant in the corresponding treatment group. More details on the sham neurofeedback setup and the real-time fMRI setup are provided in the following paragraphs.

Similar to our previous study, we used shaping in both cases in order to facilitate learning and motivation (Weiskopf *et al.*, 2004; Linden *et al.*, 2012; Papoutsi *et al.*, 2018), whereby the difficulty in increasing the height of the feedback bar was adjusted according to the participants’ performance in the preceding block.

### Target ROI selection

The NFT target ROIs were drawn at the start of each NFT session using Turbo-Brain Voyager. For the activity NFT sessions, the SMA was selected as the target ROI. For the connectivity NFT sessions, the SMA and the left striatum (including putamen, globus pallidus and caudate) were selected as the target ROIs. Similar to our previous study (Papoutsi *et al.*, 2018) and comparable to other studies (Subramanian *et al.*, 2011; Paret *et al.*, 2014, 2016; Nicholson *et al.*, 2017), the ROIs were re-drawn at each session ensuring that only voxels with high activation are selected. The ROIs from the first visit were used as a reference, when drawing the ROIs for the subsequent visits to ensure that the position was similar, although the exact voxels selected might be different. For the SMA, the statistical map was thresholded at *t*-value = 3 and a rectangle was drawn around the SMA cluster for the active versus rest contrast. The mean (SD) number of voxels for the SMA ROI was 207 (82) and 156 (51) voxels for the activity and connectivity groups, respectively. The mean (SD) size of the rectangles drawn was 8.5 (1.7) × 10.3 (3.1) × 9 and 7.7 (1.4) × 8.8 (1.7) × 9 voxels for the activity and connectivity groups, respectively. There was a significant difference between the two groups in the size of the ROI (two-sample *t*-test *t*(30) = 2.12, *P* = 0.042). This was incidental, since the ROIs were drawn around significant clusters during the localizer.

**Figure 2:**
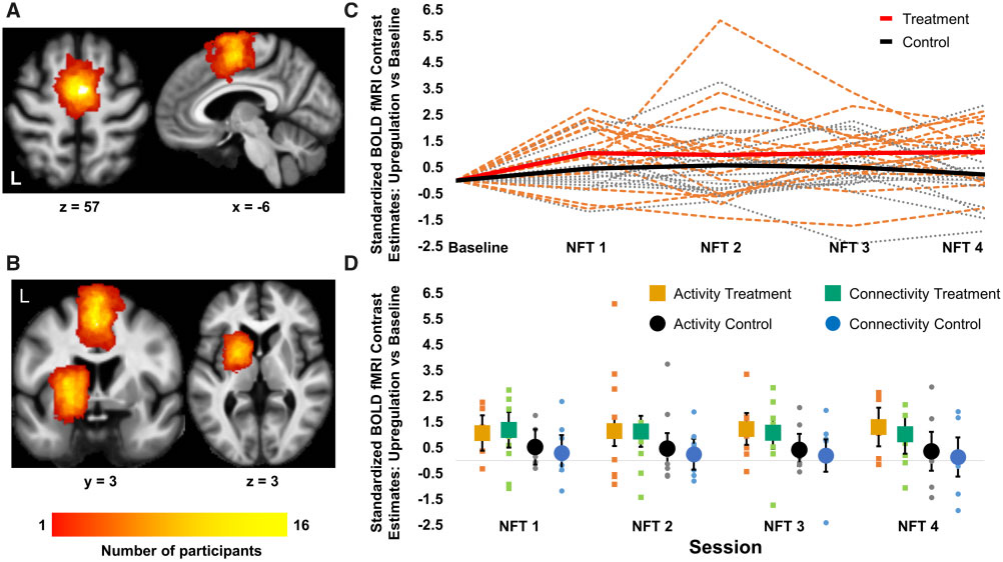
**Learning effects in activity and connectivity NFT.** (**A** and **B**) Heat maps showing the location and overlap of the target ROI across all participants in the activity and connectivity NFT groups, respectively. Maps are superimposed on a group average MT image. (**C**) Change from baseline in the target NFT levels across all training sessions per subject (dotted lines). The group mean per session is shown with thick continuous lines. Shown in red (group mean) and orange (individual participants) is the treatment group, whereas shown in black (group mean) and grey (individuals) is the control group collapsed across both types of NFT. (**D**) Dot plots show the change in NFT target levels from baseline across all NFT sessions for the four subgroups: activity treatment group (orange squares), connectivity treatment group (green squares), activity control group (black circles) and connectivity control group (blue circles). The horizontal grey line in the dot plots shows the baseline, data points above this line represent an increase compared to baseline. The small squares and circles are the individual data points, whereas the larger squares and circles show the adjusted mean group effects. Error bars are 95% CI.

The location of the striatum was identified visually on the first echo-planar imaging (EPI) scan of the localizer run using landmarks and the EPI contrast. Due to high iron concentration, the putamen and globus pallidus appear darker on an EPI scan and are therefore easy to identify on EPI scans. A rectangle was drawn around the striatum including the putamen, globus pallidus, caudate and ventral striatum. Because of the rectangular shape, the striatal ROI, also included surrounding white matter. However, the ROI was centred around the striatum and most of the recorded signal originated from the grey matter of the striatum. The mean (SD) number of voxels for the striatal ROI was 92 (83) and the mean (SD) size of the rectangle drawn was 4.9 (0.7) × 7.1 (1.5) × 9 voxels. A heat map showing the overlap of the ROIs across all participants is shown in Fig. 2A and B. We chose to define our ROIs using a functional localizer and anatomical landmarks, rather than creating an anatomical mask, because it was not always possible to acquire a structural MRI volume during the baseline visit due to patient fatigue.

### Sham neurofeedback

We chose to use a sham neurofeedback for the control groups in order to control for potential placebo effects as a result of recruiting participants to an interventional study (Foroughi *et al.*, 2016; Thibault *et al.*, 2016; Sorger *et al.*, 2019). By choosing the ‘yoked’ approach, we ensured that the feedback control participants received was biologically plausible and matched to that of the treatment group. We chose not to use the approach of using a different ROI for the control group, because of potential problems with the spread of training effects across other brain regions. We do not yet understand the mechanism underlying NFT in Huntington’s disease and how widespread any effects could be, therefore, we were not certain which other regions in the brain would be appropriate to use as control targets (Mehler *et al.*, 2018). To confirm whether the feedback received by the participants was contingent to their own brain activity, we performed confirmatory analyses after the end of the study and found that the correlation between the control participants’ true BOLD signal and the BOLD signal of their yoked participant from the treatment group was very low (see Supplementary methods).

### Data processing and analyses

#### MRI acquisition parameters

All scanning was performed on a Siemens TIM Trio 3T scanner using a standard 32-channel head coil. For the fMRI tasks, we used a whole-brain multi-shot 3D EPI sequence (Lutti *et al.*, 2013) with TR = 1.2 s, TE = 30 ms, excitation flip angle = 15°, bandwidth = 2604 Hz/Px. There were 60 slices per slab, acquired with sagittal orientation and anterior to posterior phase encoding. Image in-plane resolution was 64 × 64 and voxel size = 3 × 3 × 3 mm^3^. To allow fast whole-brain coverage, we used GRAPPA parallel imaging in phase encoding and partition encoding direction with 2 × 3 acceleration. Quantitative Multi-Parameter Maps (Draganski *et al.*, 2011; Weiskopf *et al.*, 2013; Callaghan *et al.*, 2014) and diffusion weighted imaging (DWI) scans were also acquired during the baseline and three follow-up sessions. The acquisition details are included in the Supplementary methods. Because we did not find any significant differences in the fMRI data to suggest successful training and transfer, we did not proceed with the statistical analysis of the MPMs and DWI images.

##### Real-time fMRI setup

For the NFT sessions, the EPI volumes were exported using Ice and Gadgetron (Hansen and Sørensen, 2013). In-house scripts created using Gadgetron and MATLAB (Mathworks) were used to reconstruct the 3D EPI data using SENSE (Pruessmann *et al.*, 1999) such that they could be read in near real time by Turbo-Brain Voyager to produce the target ROI time-series. There was a small delay at the start of each run to enable MATLAB to start, but after about 15 s both the MRI scanner and the Gadgetron pipeline were fully in-synch with ∼1 s latency. To enable both systems to synchronize we introduced a delay of 18 volumes at the start of each run. During that time participants viewed a white cross on a black background followed by a count-down (from 10 to 1) until the NFT paradigm started. In-house MATLAB scripts were used to process the ROI time-series and record participants’ responses, breathing and heart rate. For the NFT runs, the ROI signal was regressed against head motion traces and physiological noise from respiration (Birn *et al.*, 2008) and cardiac rhythm using RETROICOR (Glover *et al.*, 2000). The ‘cleaned’ signal was then processed by in-house MATLAB scripts using Cogent toolbox (http://www.vislab.ucl.ac.uk/cogent_2000.php, accessed 28 April 2020) to calculate and present the feedback to the participant. The computer setup in the scanner is shown in Supplementary Fig. 1B.

#### Data processing

All statistical analyses were performed after extensive quality control and offline pre-processing of the fMRI data. Supplementary Figs 3 and 4 show the evoked response patterns and average correlation coefficients, respectively, from the real-time processing pipeline. No statistical analyses were performed on these data, but are presented here for completeness. Statistical Parametric Mapping SPM12 (WCHN, London) was used for offline pre-processing of the fMRI data. The first three volumes were removed from all fMRI time-series apart from the NFT runs, where we removed the first 18 volumes. The images were then corrected for head motion with rigid-body realignment using a two-step approach.

For the ROI analyses the re-aligned images were smoothed in native space using an isotropic 8 mm FWHM Gaussian smoothing kernel. First-level, within-subject models included the condition of interest and noise regressors. We used two regressors modelling the upregulation and response (feedback blocks in the case of connectivity NFT) blocks for the baseline, NFT and transfer runs, and one regressor modelling the fist-clenching blocks for the localizer runs. The baseline condition was modelled implicitly. In addition, first-level models included six head motion parameter regressors produced by SPM and extracted from the PMCS (where applicable) with their temporal derivatives, the quadratic expansions of the movement parameters and their derivatives (Friston *et al.*, 1996; Ciric *et al.*, 2017), spike regressors (see Supplementary materials; Lemieux *et al.*, 2007), as well as 13 physiological noise regressors modelling the heart rate using RETROICOR and respiration (Glover *et al.*, 2000; Birn *et al.*, 2008; Hutton *et al.*, 2011; Misaki *et al.*, 2015). Temporal autocorrelation was modelled using SPM’s first-level autoregressive process (AR(1)) and a high-pass filter with 128 s cut-off.

For the activity NFT group, contrast values for upregulation versus baseline were extracted for the target ROI for each session and the highest 10% of *t*-values (Todd *et al.*, 2017) were used to calculate the average ROI value. For the connectivity NFT group, the time-series for the target ROIs (SMA and striatum) was extracted using a 6 mm sphere centred on the peak for upregulation versus baseline across all runs. The Pearson’s correlation coefficient of the time-series between the two ROIs within the upregulation periods was then calculated and transformed into Fisher z-scores.

#### Statistical analyses

Because the two NFT approaches use a different feedback measure, i.e. contrast estimates in the case of activity NFT and correlation coefficients in the case of connectivity NFT, we converted the activity and connectivity estimates to standardized scores in order to be able to compare them directly. In more detail, the SMA activity estimates and Fisher transformed SMA-striatum correlation coefficients were converted into z-scores using the mean and standard deviation from the baseline fMRI runs in the activity and connectivity NFT groups, respectively. The standardized activity and connectivity NFT target estimates were then used as outcomes in repeated-measures analyses of covariance (ANCOVAs) with group (treatment versus control), NFT type (activity versus connectivity), session and their interactions as fixed effects. Baseline level of the NFT target and its interaction with NFT type were included as covariates in all analyses to increase model sensitivity (Dimitrov and Rumrill, 2003). Session was modelled as a repeated factor within subjects. The primary endpoints for this study were NFT learning and near transfer. For the analyses testing for learning, session was modelled as a numerical factor, increasing from 0 to 3, to test for a linear increase across the training sessions. For the analyses testing for transfer effects, session was modelled as a categorical factor. Intersession covariance was modelled using heterogeneous compound symmetry, as this gave a reasonable approximation of the observed within-subject covariance while using minimal degrees of freedom. Model residuals were visually inspected using Q–Q plots and histograms for outliers and to ensure residuals meet normality assumptions. We used SAS 9.4 mixed approach to estimate the ANCOVAs. Because we used standardized measures for all the analyses, the model estimates provided are in units of standard deviation.

For the exploratory ROI analyses testing the relationship between learning and self-regulation ability in the NFT target levels with behavioural change we used between-group ANCOVAs. To extract the learning slope per participant we re-fitted the repeated-measure ANCOVA described above specifying random slope and intercept. As a measure of self-regulation ability we used the difference in NFT target level at the first and third follow-up sessions during upregulation without feedback compared to baseline. Other factors included in these models were group, NFT type and their interactions, as well as the baseline measure of cognitive and psychomotor function using the composite score. The dependent variable was the composite score at the first and third follow-up session. All tests were two-tailed and the alpha level used to determine significance was *P* < 0.05.

### Data availability

All data are available from the authors. Raw data cannot become publicly available due to lack of consent from the study participants.

## Results

### Learning effects: increase across training sessions

To examine differences in NFT learning between the treatment and control groups and the two different types of NFT we used repeated-measures ANCOVA testing for between-group differences across all NFT sessions, as well as a linear increase in the target NFT measure (Hellrung *et al.*, 2018) across sessions. The dependent variable was the standardized NFT target estimates and the model included as factors session (modelled as a continuous variable), group (treatment versus control), NFT type (activity versus connectivity) and all their interactions. The model was also adjusted for baseline NFT target levels and its interaction with NFT type. The main effect of group tests for differences in the change from baseline between the treatment and control groups across all visits, whereas the group by session tests for differences in learning slope between groups. To test the main effect of group across all training sessions we used least square mean testing and compared the NFT target estimates across all NFT sessions between treatment and control groups.

There was no evidence for a difference between the activity and connectivity treatment groups in learning slope (*P* > 0.6 for both group by NFT type and group by NFT by session interactions. Linear increase across sessions estimates 95% CI: activity treatment = 0.078 (−0.190, 0.345); connectivity treatment = −0.056 (−0.323, 0.211); activity control = −0.057 (−0.324, 0.210); connectivity control = −0.519 (−0.319, 0.215); Fig. 2C). However, there was a significant main effect of group, where the treatment group had greater NFT target levels overall compared to the control group across all visits (*t*(29.1) = 2.79, *P* = 0.009). Estimate 95% CI of group difference across all sessions: 0.816 (0.22, 1.41). The unit of the estimates is standard deviations; Fig. 2D). At baseline, there was no difference in NFT target levels between the groups (*F*(1,28) = 0.20, *P* = 0.655; see Supplementary materials), our findings therefore suggest that the effects of receiving neurofeedback occurred within the first training session and were stable across training sessions.

### Near transfer: upregulation without feedback

After the four training sessions, participants returned for three follow-up sessions. During those sessions, we examined the ability of the participants to self-regulate the NFT target levels without receiving any feedback (i.e. near transfer). Similar to our previous analyses examining learning effects, we used a repeated-measures ANCOVA with factors group, session (modelled as a categorical factor in this model), NFT type and their interactions, adjusting for the baseline NFT target levels and its interaction with NFT type. The dependent variable was the standardized NFT target contrast estimates (upregulation without feedback compared to no upregulation). In this case, we did not hypothesize any difference between sessions and expected that transfer effects would remain stable for the three follow-up visits. Therefore, the effects of interest were the main effect of group (treatment versus control), which tested between-group differences in the increase of the NFT target levels across all follow-up visits from baseline, and the group by NFT type interaction (treatment versus control by activity versus connectivity NFT).

**Figure 3:**
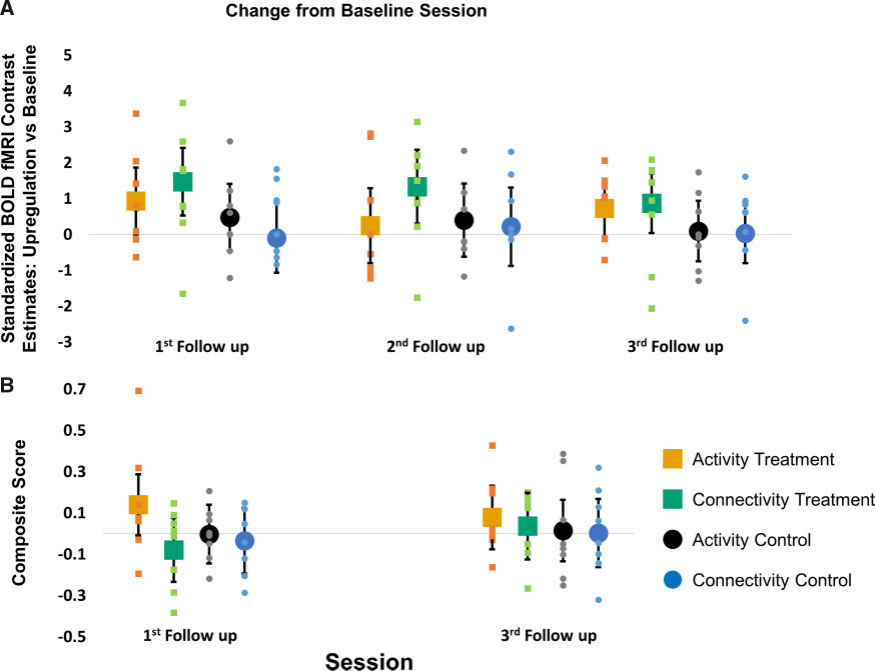
**Near and far transfer effects.** (**A**) Dot plots show the change in NFT target levels from baseline across the three follow-up sessions for the four subgroups: activity treatment group (orange squares), connectivity treatment group (green squares), activity control group (black circles) and connectivity control group (blue circles). (**B**) Dot plots show the change in the behavioural composite score from baseline across the two follow-up sessions for the four subgroups (same colour coding as above). The horizontal grey lines in both plots show the baseline, data points above this line represent an increase compared to baseline. The small squares and circles show the individual data points, whereas the larger squares and circles show the adjusted mean group effects. Error bars are 95% CI.

Although the treatment group increased NFT target levels at the follow-up visits compared to baseline by 0.9 standard deviations (estimate 95% CI increase from the baseline session: treatment group = 0.929 (0.341, 1.518); control group = 0.186 (−0.407, 0.778)), this increase was not significantly different from the control group (*F*(1,26.4) = 3.27, *P* = 0.082. Estimate 95% CI of the group difference across all sessions = 0.74 (−0.10, 1.59); Fig. 3A). The connectivity treatment group was the only group able to increase its NFT target levels at follow-up compared to baseline (estimate 95% CI increase from baseline across all follow-up sessions: connectivity treatment = 1.226 (0.389, 2.063); activity treatment = 0.633 (−0.195, 1.460); activity control = 0.322 (−0.505, 1.149); connectivity control = 0.0492 (−0.799, 0.897); Fig. 3A). However, the interaction between NFT type and group was not significant (*F*(1,26.4) = 1.11, *P* = 0.30). There were no other significant effects or interactions (all *P* > 0.29). Our results suggest that although there is some evidence regarding a near transfer effect in the treatment group, particularly in the connectivity treatment group, it is weak and not significantly better than the control group.

### Far transfer: cognitive and psychomotor performance

To assess the effect that NFT had on participants*’* performance in tasks unrelated to the training (far transfer), we examined change from baseline after training in the composite score comprising of measures previously shown to be sensitive to Huntington’s disease progression (Tabrizi *et al.*, 2011; Papoutsi *et al.*, 2018). We performed a similar mixed linear model analysis to the one described in the near transfer section above. The cognitive composite score was the dependent variable and the model was adjusted for the baseline level of the cognitive composite score. The effects of interest were the main effect of group and the group by NFT type interaction, which test for between*-*group differences in change from baseline across all the two follow-up sessions.

Although the difference between treatment and control groups is in favour of the treatment group (estimate 95% CI increase from baseline: treatment = 0.044 (−0.059, 0.146); control = −0.005 (−0.102, 0.091)), it is not significant (*F*(1,27) = 0.63, *P* = 0.435) and the magnitude of the change is small (estimate 95% CI difference = 0.049 (−0.077, 0.175) standard deviations). There was also no evidence for a difference between the activity and connectivity treatment groups (group by NFT type interaction *F*(1,27) = 0.76, *P* = 0.39). Estimate 95% CI increase from baseline: activity treatment group = 0.108 (−0.023, 0.240); connectivity treatment = −0.022 (−0.162, 0.119); activity control = 0.006 (−0.120, 0.131); connectivity control = −0.016 (−0.161, 0.130); Fig. 3B). There were no other significant effects or interactions (all *P* > 0.18). Our results therefore do not provide any evidence for a significant far transfer effect of NFT.

A detailed description of the average change in the individual scores that comprised the composite score is presented in the Supplementary materials and can provide more insight on the magnitude of change in the individual tests (Supplementary Fig. 5 and Table 3).

### Association with change in performance

As an exploratory analysis, we examined whether NFT-related measures, specifically learning slope and/or self-regulation ability (near transfer), predict improvement in the cognitive composite score. If successful upregulation has an effect on behaviour than we should see a relationship between increasing ones NFT target levels and improvement in behaviour after training.

We first tested the relationship between training slope, i.e. change from the first to the last NFT training session, and behavioural performance at the first follow-up session, i.e. within 2 weeks from the end of training. The NFT learning slope for each participant was extracted from a random slope and random intercept mixed linear model testing for linear increase in NFT target levels across visits. To test the effect of NFT learning on behaviour, we used an ANCOVA with factors NFT target level slope (linear increase across NFT sessions), group (treatment vsersu control), NF type (activity versus connectivity) and their interactions. The model was also adjusted for the cognitive score at baseline and the dependent variable was the composite score at the first follow-up. The effects of interest were the main effect of learning slope and its interactions with group and NFT type. The main effect of learning slope tests whether there is a relationship between increase in NFT slope across training sessions and improvement in behaviour at follow-up across all groups. The interactions between learning slope and group (and NFT type), test whether there is a difference in the relationship between improvement in behaviour across the different groups.

**Figure 4:**
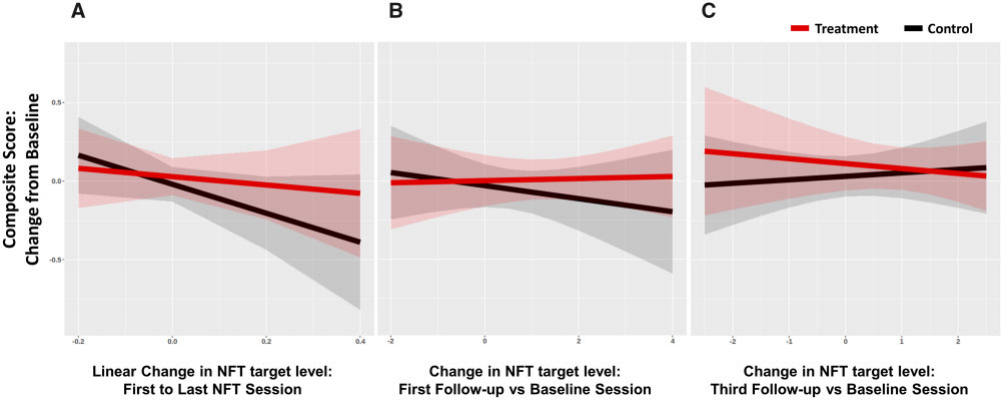
**Relationship between change in the composite score and change in NFT target levels.** (**A**) Regression lines plot the relationship between change in the composite score at the first follow-up from baseline and change in NFT target levels from the first to the last NFT training visit adjusted for baseline levels. Shown in **B** is the relationship between change in the composite score at the first follow-up from baseline and change in NFT target levels at the first follow-up session compared to baseline. Shown in **C** is the relationship between the same measures as in **B**, but for the third follow-up. Regression lines and 95% CI for the treatment (red) and sham control (black) groups are averaged across both NFT type groups.

The relationship between NFT learning slope across all groups and change in the composite score was not significant (*F*(1,23) = 2.67, *P* = 0.12) and negative (estimate 95% CI of change in the composite score per unit increase in slope = −0.59 (−1.35, 0.16)). There was also no significant difference between treatment and control groups in the relationship between NFT slope and composite score change (*F*(1,23) = 0.89, *P* = 0.36; estimate 95% CI of the between-group difference = 0.71 (−0.74, 2.15)), or evidence of a positive relationship in the treatment group (estimate 95% CI of change in the composite score per unit increase in slope for the treatment group = −0.24 (−1.24, 0.78); control group = −0.94 (−2.00, 0.12); Fig. 4A). All other effects and interactions were also non-significant (all *P* > 0.1).

We then tested the relationship between volitional NFT target upregulation ability and improvement in the composite score at follow-up (first and third follow-up separately). We used ANCOVA with factors NFT target level estimate (the difference between NFT target contrast estimates at each follow-up versus baseline), group (treatment versus control), NF type (activity versus connectivity) and their interactions. The model was adjusted for baseline performance in the composite score and the dependent variable was the composite score at the first and third follow-up. The effects of interest were the relationship between behavioural change at follow-up and self-regulation ability, as well as the interaction with group and NFT type.

The relationship between NFT target upregulation ability across all groups and change in the composite score was not significant for either of the follow-ups (first follow-up: *F*(1,22) = 0.28, *P* = 0.60; third follow-up: *F*(1,22) = 0.02, *P* = 0.90) and almost zero in both cases (estimate 95% CI of change in the composite score per unit increase in slope for the first follow-up = −0.02 (−0.09, 0.05) and for the third follow-up = −0.01 (−0.08, 0.07)). There was also no significant difference between treatment and control groups in the relationship between NFT slope and composite score change (first follow-up: *F*(1,22) = 0.56, *P* = 0.46; estimate 95% CI of the between-group difference = 0.08 (−0.09, 0.25); third follow-up: *F*(1,22) = 0.48, *P* = 0.50; estimate 95% CI of the between-group difference = 0.03 (−0.16, 0.22); Fig. 4B and C). All other effects and interactions were also non-significant (all *P* > 0.1).

## Discussion

The present proof-of-principle study examined the use of NFT for the treatment of cognitive and psychomotor impairment in Huntington’s disease patients. For this purpose, we used two different NFT approaches (activity and connectivity) in a single-blind, randomized controlled trial study, with yoked sham NFT control groups, an intensive training protocol consisting of 16 NFT trials over four sessions, optimized real-time fMRI acquisition protocol and using objective, a priori defined, measures of cognitive and psychomotor function. This enabled us to collect rigorous evidence regarding the usefulness of NFT in treating cognitive and psychomotor symptoms in Huntington’s disease. We found strong evidence of a difference between treatment and control groups during the NFT sessions, such that participants in the treatment group increased the levels of the NFT target more than participants in the control group, when receiving NFT. However, evidence regarding the ability of the participants to volitionally upregulate their NFT target levels after training was weak. It is therefore unclear whether participants learned to regulate their brain activity and were able to apply the learning in the absence of NFT. We also did not find robust evidence of improvement in cognitive and psychomotor function after training in the treatment group, or of a relationship between NFT learning and change in cognitive and psychomotor function.

In more detail, we found a significant difference between treatment and control groups in terms of the increase of their NFT target levels from baseline. Participants in the treatment group increased their activity and connectivity levels from baseline by 0.74 standard deviations more than participants in the sham control group. This difference was present from the first training session until the last and we did not observe any further increase in the subsequent training sessions. This finding is in agreement with previous studies which have shown that participants can learn to regulate the target NFT levels within one visit (Hellrung *et al.*, 2018; Kohl *et al.*, 2019).

It is important to note that our study was single-blind, it is therefore possible that the difference observed between treatment and control groups could have been because of unconscious researcher bias. We believe that this is unlikely, since the participants were in the MRI scanner during NFT and they had minimal contact with the researchers. In addition, if they were such effects, we would expect that they would have been more pronounced during cognitive and psychomotor testing, during which the researchers had longer contact with the participants. However, we did not find any evidence for a difference between the two groups, treatment and controls. Therefore, we believe that the measured difference between the two groups during NFT training reflects the effect of providing feedback to the participants on their NFT levels and them adjusting their behaviour accordingly.

After NFT we tested participants’ ability to increase the levels of the NFT target without receiving neurofeedback. This way we can test whether participants have truly learned to regulate the levels of the NFT target and are therefore able to upregulate without receiving NFT. Although the treatment group increased their NFT target levels from baseline at follow-up by 0.9 standard deviations, this increase was not significantly different from the control group. Our results therefore suggest that although there is some evidence regarding a near transfer effect in the treatment group, particularly in the connectivity treatment group, it is weak and at present ambiguous and requires replication.

Furthermore, we did not find robust evidence of improvement in cognitive and psychomotor function after NFT. To measure cognitive and psychomotor function, we used a composite score comprised of a priori identified, objective measures of cognitive and psychomotor function, sensitive to Huntington’s disease progression. However, our study was powered to detect differences in near transfer, not far transfer, therefore it is possible that we were underpowered to detect significant group differences in behaviour. Analyses of the cognitive and psychomotor measures were exploratory, but important to include in order to guide the design of future studies for clinical efficacy. Although the between-group difference in the change from baseline was in favour of the treatment group, the magnitude of the improvement was very small and clinically non-significant representing a change of 0.05 standard deviations in the composite score. We also did not find any evidence that change in NFT-related measures, specifically NFT learning slope and ability to self-regulate, related to change in behaviour.

It is possible that by using a composite score we obscured large effects in specific measures. In addition to the composite score, we therefore also reported change in the individual measures (Supplementary Table 3 and Fig. 5). The Stroop word reading task shows a large increase in the first follow-up (mean (SD) = 5.75 (4.7)) in the activity NFT treatment group. This is in agreement with the results reported in our previous work (Papoutsi *et al.*, 2018), where we also show a large improvement in this measure. However, this is a *post hoc* finding and would need to be verified in future research.

Taken together our findings are in agreement with recent studies that showed differences between treatment and controls groups during NFT, but weak evidence for near or far transfer (Schabus *et al.*, 2017). The failure to find reliable evidence of clinical benefit could be because we did not target the right regions or connections. In this study, we used two different NFT targets, SMA activity and SMA-striatum connectivity. The latter was selected based on our previous work which showed that improvement in cognitive and psychomotor function predicted increased SMA-striatum connectivity across NFT sessions (Papoutsi *et al.*, 2018). In the present study, despite using the same cognitive and psychomotor measures as in our previous study and targeting the networks identified in our previous study, we did not find any evidence to suggest that SMA-striatum connectivity NFT relates to improvements in cognitive and psychomotor function. Therefore, we were not able to replicate this finding from our previous work. It is possible that other regions and connections, such as the left inferior parietal lobe, which have been implicated in neural compensation in Huntington’s disease (Klöppel *et al.*, 2015), could have been more appropriate. This remains to be tested.

Finally, in our study we did not find strong evidence to suggest a difference between the two NFT methods, activity and connectivity. The connectivity treatment group was able to increase the NFT target levels at follow-up compared to baseline; however, there were no significant group differences. Although targeting SMA-striatal connectivity is theoretically motivated by knowledge regarding the disease mechanism and was identified as a potential target in our previous study (Papoutsi *et al.*, 2018), we did not find any reliable evidence that it was better or worse than activity NFT. Both activity (Young *et al.*, 2017; Hellrung *et al.*, 2018) and connectivity (Megumi *et al.*, 2015; Ramot *et al.*, 2017; Yamashita *et al.*, 2017) NFT have been used successfully in other studies, suggesting that both methods are effective.

A limitation of our study is that we could not dissociate the use of connectivity NFT from the use of intermittent feedback. In our study, feedback was provided continuously in near real time in the activity NFT group, whereas in the case of connectivity NFT, correlations were computed over 30 s and the feedback was presented intermittently at the end of the upregulation block. Therefore, the two elements, frequency of feedback presentation and NFT type, were intertwined and could not be separated. A previous study comparing continuous versus intermittent feedback using per cent signal change in the amygdala in healthy young adults showed that participants were able to learn to increase the target NFT levels using both approaches, although intermittent feedback was more effective than continuous in that study (Hellrung *et al.*, 2018). In our study, we did not find any evidence for a difference between the two approaches.

To conclude, in the present study we compared two different NFT approaches in Huntington’s disease, SMA activity and SMA—left striatum connectivity NFT against sham NFT control groups, in terms of learning and transfer. We used a randomized controlled trial design and an intense, optimized real-time fMRI NFT protocol, to ensure that we can acquire rigorous evidence regarding the role of real-time NFT in Huntington’s disease. Our findings support previous claims that using NFT participants can be guided to increase their levels of cortical activity and cortico-striatal connectivity using real-time fMRI NFT. However, evidence regarding the transfer of learning to volitional control of brain activity and behaviour are promising, but at present weak.

## Supplementary Material

fcaa049_Supplementary_Data

## Abbreviations

ANCOVA =: analysis of covariance;
BOLD =: blood oxygen level dependent;
CAP =: CAG by age product;
CI =: confidence interval;
DWI =: diffusion weighted imaging;
EPI =: echo-planar imaging;
fMRI =: functional MRI;
FWHM =: full width at half maximum;
GRAPPA =: generalized autocalibrating partial parallel acquisition;
HADS =: Hamilton anxiety and depression score;
HRF =: Hemodynamic Response Function;
HTT =: Huntingtin;
MoCA =: Montreal cognitive assessment;
MPM =: multi-parameter maps;
NFT =: neurofeedback training;
PMCS =: prospective motion correction system;
ROI =: region of interest;
SD =: standard deviation;
SDMT =: Symbol Digit Modalities Test;
SMA =: supplementary motor area;
SPM =: statistical parametric mapping;
TFC =: total functional capacity;
TMS =: Total Motor Score;
UHDRS =: Unified Huntington’s Disease Rating Scale;
WCHN =: Wellcome Centre for Human Neuroimaging
