# Supplementary material for: Activity or connectivity? A randomized controlled feasibility study evaluating neurofeedback training in Huntington’s disease
Source: Brain Commun. 2020 Apr 23;2(1):fcaa049. doi: 10.1093/braincomms/fcaa049 (PMC7425518; doi:10.1093/braincomms/fcaa049)
Supplement: fcaa049_Supplementary_Data [file Original_submission_decision_letter.pdf › Original_submission_decision_letter.pdf]

BRAINCOM-2019-124
23-Nov-2019

Dear Dr. Papoutsi,

Thank you for your recent submission BRAINCOM-2019-124
entitled "Activity or Connectivity? Evaluating neurofeedback training in Huntington's disease" to Brain Communications.

Your manuscript has been reviewed, and while the reviewers had largely positive comments, they have some constructive critiques that I think you could address in a minor revision of the manuscript. Therefore, I invite you to respond to the reviewer(s)' comments and revise your manuscript. The reviewer(s)' comments are included at the bottom of this message.

In addition to the reviewers' comments, please note our editorial comments added below to comply with our requirements for presentation of scientific data at Brain Communications.

Please also note that for the final accepted paper, we will require an abbreviated summary of no more than 50 words and a graphical abstract. Please include these in your revision.

Please format your revised manuscript according to our Instructions to Authors https://academic.oup.com/braincomms/pages/General_Instructions – a summary of key points is attached to help with this.

To revise your manuscript, click the link below or log into https://mc.manuscriptcentral.com/braincom and enter your Author Centre, where you will find your manuscript title listed under "Manuscripts with Decisions." Under "Actions," click on "Create a Revision." Your manuscript number has been appended to denote a revision.

*** PLEASE NOTE: This is a two-step process. After clicking on the link, you will be directed to a webpage to confirm. ***

https://mc.manuscriptcentral.com/braincom?URL_MASK=6d64de1fb0654ae0bde9405935256466

Please upload a revised manuscript with the changes you have made shown either with "track changes" or in coloured text.

When submitting your revised manuscript, you will be asked to respond to the reviewer(s) comments. Please be as specific as possible in your response to all of the comments.

The editorial team at Brain Communications also aims to increase transparency in scientific publishing and to demonstrate the value that peer review brings to the scientific process. Please therefore consider whether you are willing to have reviewer comments on your paper and your response published as supplementary data with the paper if it is accepted. This is entirely optional and will only be done if both the authors and reviewers agree. When submitting your revision you will have the opportunity to update the answer you entered previously.

IMPORTANT: Your original files are available to you when you upload your revised manuscript. Please delete any redundant files before completing the submission.

Because we are trying to facilitate timely publication of manuscripts submitted to Brain Communications, we would appreciate your revised manuscript as soon as possible. If it is not possible for you to resubmit within 3 months, the option to submit a revision will expire, and we would then consider your revised paper as a new submission. However, if you need a little more time, please get in touch and we can extend this deadline for you.

Once again, thank you for submitting your manuscript to Brain Communications and I look forward to receiving your revision.

Yours sincerely,
Prof. Kevin Talbot
Editor, Brain Communications

Reviewer(s)' Comments to Author:

Reviewer: 1

Comments to the Author
In this study fMRI guided neural feedback training (NFT) is used in an attempt to train individuals with early HD to regulate their own brain activity. Overall, this is a carefully conducted study, which presents important negative data that should be published. Data is presented that demonstrates that patients can learn to regulate brain activity and that fMRI NFT aids this process, but there is only weak transfer to improvement when the feedback is removed and no evidence that the intervention leads to cognitive improvement.

I have a number of queries/recommendations:
Major:
What is the basis for expecting increased activity in SMA to improve the cognitive measures used in this study? The authors state that the cognitive composite score they used, which includes several tests commonly used in the assessment of HD patients plus a test of negative emotion recognition and inter-tap interval, is sensitive to disease progression. However, measurable progression is unlikely in such small numbers over the time over which the study is conducted. It is otherwise it is unclear what the neural basis is for expecting improvement on this specific set of measures.

Although I agree with the authors that they currently have no evidence of the value of fMRI NFT as an intervention, and so cannot recommend it based on their data, there are many reasons why no effect, other than fMRI NFT being ineffective, has been found. Many reasons are highlighted in the paper, such as the small numbers and whether SMA is the correct target area, although others, such as whether the amount of training given is insufficient (much longer training periods are needed for patients undergoing physical activity training in order to see transference effects) are not discussed or covered on less detail. Nevertheless, I believe it is premature to make a recommendation from this study alone. The statement of recommendation at the end of the abstract implies that fMRI NFT is not worthy of further follow up and I think this is premature and should be removed

Minor:
A clearer description of the NFT process in the main methods and an explanation of the purpose of teeth impressions would be helpful (this it is explained in the supplementary data but not well explained in the main methods).

P6 line 30 reference missing

Reviewer: 2

Comments to the Author
Overall, the idea behind this paper is very interesting. However, given the trends found in some results and the small sample size, the overall conclusion that NFT should not be recommended for HD seems too strong. Instead it appears that more statistical power is required for this to be properly tested. If more data is collected, or if the sample sizes used can be better proven to be appropriate, then the results would be of more interest.

Major points

The authors try to justify the small sample size (n=8 per group) based on effect sizes calculated from another paper (Yoo et al., 2008). However, this paper had a different ROI (it targeted the primary as opposed to the supplementary motor area) and, more importantly, a different population (healthy participants as compared to those with HD). The ROIs used in the current study (and their connectivity) are known to be degraded in patients with HD (the authors themselves state this) and so a study based on (different ROIs in) healthy participants does not seem appropriate for estimating statistical power in this case.

Given such a small sample size, potential effects of NFT on HD should only be ruled out if all of the participants clearly show no effect. However, the between group main effect of "near transfer" at the follow-up visits appeared to trend towards significance ($p = 0.082$). Results indicate that the treatment group (estimate 95% CI increase from the baseline session: treatment group = 0.929 (0.341, 1.518)), but not the control group (estimate 95% CI increase from the baseline session: treatment group = 0.186 (-0.407, 0.778)) may have been able to increase NFT target levels compared to baseline. This is particularly the case for the connectivity treatment group (estimate 95% CI increase from baseline across all follow-up sessions: connectivity treatment = 1.226 (0.389, 2.063)). However, this finding is basically disregarded because the interaction between NFT type and group was not significant ($F(1, 26.4) = 1.11$, $p = 0.30$). I agree with the authors' statement that this evidence is "weak and at present ambiguous". However, I think it is greatly overstated to say that these results indicate "agreement with

recent studies showing a lack of clinical benefit of NFT". Instead I wonder, particularly because the between group main effect p-value trended towards significance, if this result simply reflects a lack of statistical power as a result of the small sample size. Overall, I think it is greatly unjustified to write a paper indicating that NFT might not be suitable for treatment of HD when results show that treatment effects trend towards significance with a small sample size.

Furthermore, in the introduction of the current paper, the authors focus on the current lack of "treatments for cognitive impairment in HD. However, cognitive effects were not examined specifically, but instead combined with psychomotor effects to make a composite score. While the dangers of multiple comparisons must be considered, this composite method may actually be too conservative to show real effects, especially given (a) the small sample size, and (b) the fact that it combines the results of tasks that measure such different things; e.g. the Stroop word task versus finger tapping tasks. For example, the measure that showed the most improvement (across all patients) in the authors' 2018 paper (where activity NFT was used) appears to be the Stroop word task. In the current paper (for participants in the activity NFB condition) there again appears to be an (what looks like a significant) improvement in the Stroop work task. However, because the results of this task were combined with those of 6 other tasks, nothing of significance was found. I wonder if, had a larger sample size or a measure other than the composite score been used, the improvement in Stroop word task performance would have been found to correlate with NFT performance and/or near transfer? The authors conclude that "It is therefore doubtful whether NFT, even if it was successful, could lead to improved cognitive and psychomotor function in HD". However, I would say that based on the results (especially when the Stroop results are considered), this may again be a null finding simply due to low statistical power and due to the (composite) nature of analysis. While it is good to be conservative in statistical analyses, if there is not enough statistical power in analyses then strong conclusions about null-effects should not be made about non-significant results. Instead more samples should be collected to better test the hypothesis with more power.

Minor points

The authors acknowledge that "A limitation of our study is that we could not dissociate the use of connectivity NFT from the use of intermittent feedback" but they do not give any explanation (that I could find) as to why they decided to use continuous feedback for the activity NFT condition and intermittent feedback for the connectivity NFT condition. Furthermore, the number of rest blocks (6 x 30s for the activity NFT condition and 5 x 45s for the connectivity NFT condition), the length of rest blocks (30s for the activity NFT condition and 45s for the connectivity NFT condition), and even the length of the overall NFT sessions, differed between these conditions (6x30s + 5x18s + 5x30s = 7 mins for the activity NFT condition; and 5x45s + 5x30 +5x3 = 6.5mins for the connectivity NFT condition). No explanation for these differences in task design between the two conditions is provided, and these differences provide too many potential confounds for a proper examination of activity versus connectivity NFT (this would have been a major point if significant differences had been found).

I cannot find any information about what the "response" is in the "response blocks" of the activity NFT condition. It does say "The rest and response blocks were identical to those of the transfer runs described previously." Is this referring to the 2018 paper? This is not clear and in fact, since supplementary figure 2C shows a question mark on screen during the response blocks, it appears as if the participants need to make some behavioral response. The 2018 paper says "response blocks were 16 s to allow for the hemodynamic response to return to baseline prior to the upregulation blocks". If the response blocks in the current paper function in the same way then this is understandable that this is necessary due to the continuous nature of the feedback in the activity NFT condition. However, why are response blocks 16s long in the 2018 paper and 18s in the current paper? And why there is no control for the presented stimulus (a question mark on the screen) in the connectivity NFT condition where feedback is intermittent? A better explanation of what the response blocks entail is needed.

On average, how many voxels were included in each ROI? What was the size of the rectangle drawn around each?

Why weren't the analyses adjusted for caudate volume atrophy like they were in the 2018 paper?

Editorial Office Comments:
-the appropriate checklist must be included (see Reporting Guideline section within 'Instructions to authors').
-If your paper is accepted, we will require an abbreviated summary of no more than 50 words and a graphical abstract. Please include these in your revision.
