# Supplementary material for: Activity or connectivity? A randomized controlled feasibility study evaluating neurofeedback training in Huntington’s disease
Source: Brain Commun. 2020 Apr 23;2(1):fcaa049. doi: 10.1093/braincomms/fcaa049 (PMC7425518; doi:10.1093/braincomms/fcaa049)
Supplement: fcaa049_Supplementary_Data [file Original_submission_response_to_reviewers.pdf › Original_submission_response_to_reviewers.pdf]

HUNTINGTON'S DISEASE CENTRE

Department of Neurodegenerative Disease
Join Head of Department:
Professor Sarah J Tabrizi FRCP PhD FMedSci

Marina Papoutsi PhD
Senior Research Fellow
UCL Huntington's Disease Centre
Tel: +44 (0)20 3108 7478
Email: m.papoutsi@ucl.ac.uk

Professor Kevin Talbot
Brain Communications Editor
Ormond House
27 Boswell Street
London WC1N 3JZ

10th February 2020

Dear Professor Talbot,

Re: Activity or Connectivity? A randomized controlled feasibility study evaluating neurofeedback training in Huntington's disease. Papoutsi et al.

We are grateful for your consideration of our manuscript and to the reviewers for the helpful comments.

In the following pages we provide our response to the reviewers' comments. Our response is written with red ink. Excerpts copied from the manuscript are in quotes and written in italics.

As suggested by the editorial office, we have included the CONSORT checklist for pilot and feasibility studies and made edits to ensure compliance with the recommendations, where the requested information was missing.

All revisions made to the original submitted manuscript are highlighted in yellow.

Many thanks for considering our manuscript.

Yours sincerely,

Marina Papoutsi

Reviewer: 1

In this study fMRI guided neural feedback training (NFT) is used in an attempt to train individuals with early HD to regulate their own brain activity. Overall, this is a carefully conducted study, which presents important negative data that should be published. Data is presented that demonstrates that patients can learn to regulate brain activity and that fMRI NFT aids this process, but there is only weak transfer to improvement when the feedback is removed and no evidence that the intervention leads to cognitive improvement.

I have a number of queries/recommendations:

1. What is the basis for expecting increased activity in SMA to improve the cognitive measures used in this study? The authors state that the cognitive composite score they used, which includes several tests commonly used in the assessment of HD patients plus a test of negative emotion recognition and inter-tap interval, is sensitive to disease progression. However, measurable progression is unlikely in such small numbers over the time over which the study is conducted. It is otherwise it is unclear what the neural basis is for expecting improvement on this specific set of measures.

   We thank the reviewer for the opportunity to clarify these points. The reviewer raises an important issue: given that measurable progression in such small numbers and such a short time period is unlikely, what is the basis for expecting improvement on this set of measures using NFT?

   The cognitive and psychomotor measures used in this study were selected because they are objective measures, sensitive to HD cognitive and psychomotor impairment, they are therefore clinically relevant. We agree with the reviewer that significant behavioural change was unlikely to be achieved in such a small study and within such a short period. This is the reason why the primary endpoint of our feasibility study was near transfer, i.e. the volitional increase in the NFT target measures after training, and not change in the behavioural measures, i.e. far transfer. However, we hope that the reviewer agrees with us that reporting the effect of the intervention on behavioural measures is important even in a feasibility study. The main reason is that it provides an estimate of predicted effect size that can be used to power future RCTs designed to test the efficacy of the intervention. We refer to this point in the manuscript pp.5-6.

   > *"As this was a feasibility study, we chose to power on near transfer and not far transfer effects. Although near transfer effects are not clinically relevant, they do allow us to test NFT learning transfer and as such can serve as a suitable endpoint for this feasibility study. If the findings from this study are promising, then the effect sizes estimated from this study could be used to power a future RCT focusing on efficacy".*

   In addition, we have emphasized this point in the discussion, p.18.

   > "*However, our study was powered to detect differences in near transfer, not far transfer, therefore it is possible that we were underpowered to detect significant group differences in behaviour. Analyses of the cognitive and psychomotor measures were exploratory, but important to include in order to guide the design of future studies for clinical efficacy*".

2. Although I agree with the authors that they currently have no evidence of the value of fMRI NFT as an intervention, and so cannot recommend it based on their data, there are many reasons why no effect, other than fMRI NFT being ineffective, has been found. Many reasons are highlighted in the paper, such as the small numbers and whether SMA is the correct target area, although others, such as whether the amount of training given

is insufficient (much longer training periods are needed for patients undergoing physical activity training in order to see transference effects) are not discussed or covered on less detail. Nevertheless, I believe it is premature to make a recommendation from this study alone. The statement of recommendation at the end of the abstract implies that fMRI NFT is not worthy of further follow up and I think this is premature and should be removed

We thank the reviewer for the recommendation, we agree that the wording is too strong and have deleted this sentence from the manuscript on p. 19 and from the abstract (last sentence). We also toned down the concluding sentence on p. 19.

"However, evidence regarding the transfer of learning to volitional control of brain activity and behaviour are promising, but at present weak".

3. A clearer description of the NFT process in the main methods and an explanation of the purpose of teeth impressions would be helpful (this it is explained in the supplementary data but not well explained in the main methods).

   We apologise for the brevity of the methods section, this is due to the word count restrictions of the journal. We have edited the methods section on p. 6 to explain the rationale for the PMCS and the dental impressions.

   "A Prospective Motion Correction system (PMCS) was used to correct head motion during scan acquisition (Zaitsev et al., 2006; Todd et al., 2015). Using the PMCS allowed us to recruit participants with moderate chorea without substantial data loss due to head motion. Details of the PMCS are provided in the supplementary materials. Participants who consented to the use of the PMCS had teeth impressions acquired during the screening visit by a qualified orthodontist (NH). The teeth impressions were used to create a small retainer on which the optical marker for the PMCS was mounted".

   In addition, we have rewritten the section explaining the NFT runs in response to Reviewer 2's comments (point 4). Please see pp. 7-8.

4. P6 line 30 reference missing

   We have added the missing references. Thank you for bringing this to our attention.

Reviewer: 2

Overall, the idea behind this paper is very interesting. However, given the trends found in some results and the small sample size, the overall conclusion that NFT should not be recommended for HD seems too strong. Instead it appears that more statistical power is required for this to be properly tested. If more data is collected, or if the sample sizes used can be better proven to be appropriate, then the results would be of more interest.

As suggested by reviewer 1 (point 2), we have deleted and toned down the relevant parts in the abstract and conclusion (p.19). Please see our reply to reviewer 1, point 2.

Major points

1. The authors try to justify the small sample size (n=8 per group) based on effect sizes calculated from another paper (Yoo et al., 2008). However, this paper had a different ROI (it targeted the primary as opposed to the supplementary motor area) and, more importantly, a different population (healthy participants as compared to those with HD). The ROIs used in the current study (and their connectivity) are known to be degraded in patients with HD (the authors themselves state this) and so a study based on (different ROIs in) healthy participants does not seem appropriate for estimating statistical power in this case.

   We thank the reviewer for the opportunity to clarify this point. We agree with the reviewer that the Yoo et al. 2008 study we used to power our current study has limitations and indeed we acknowledge these limitations in our paper. Our study is the first RCT of NFT in Huntington's disease and one of its aims is to provide appropriate effect sizes based on our patient population for future RCTs powered for efficacy.

   The reason why we selected the Yoo et al. 2008 study at the time of designing our study is because (1) it included a sham control group, and (2) it included a near transfer comparison, i.e. the ability of the individuals to self-regulate the NFT target after NFT training. Near transfer was our primary endpoint and therefore we powered our study on the effect size calculated from the estimates provided in the Yoo et al. 2008 study. We were not able to find another more suitable study at the time of designing our study. An alternative study in Parkinson's disease patients which used the SMA as a target region (Subramanian et al., 2011) did not include a sham control group or near transfer runs and was therefore not appropriate.

2. Given such a small sample size, potential effects of NFT on HD should only be ruled out if all of the participants clearly show no effect. However, the between group main effect of "near transfer" at the follow-up visits appeared to trend towards significance ($p = 0.082$). Results indicate that the treatment group (estimate 95% CI increase from the baseline session: treatment group = 0.929 (0.341, 1.518)), but not the control group (estimate 95% CI increase from the baseline session: treatment group = 0.186 (-0.407, 0.778)) may have been able to increase NFT target levels compared to baseline. This is particularly the case for the connectivity treatment group (estimate 95% CI increase from baseline across all follow-up sessions: connectivity treatment = 1.226 (0.389, 2.063)). However, this finding is basically disregarded because the interaction between NFT type and group was not significant ($F(1, 26.4) = 1.11$, $p = 0.30$). I agree with the authors' statement that this evidence is "weak and at present ambiguous". However, I think it is greatly overstated to say that these results indicate "agreement with recent studies showing a lack of clinical benefit of NFT". Instead I wonder, particularly because the between group main effect p-value trended towards significance, if this result simply reflects a lack of statistical power as a result of the small sample size. Overall, I think it is greatly unjustified to write a paper indicating that NFT might not be suitable for treatment

of HD when results show that treatment effects trend towards significance with a small sample size.

We agree with the reviewer that a feasibility study cannot provide conclusive evidence towards clinical benefit. We have deleted and toned down the relevant parts in the abstract, the discussion (p.18) and the conclusion (p. 19). Please also refer to our reply to reviewer 1, point 2.

3. Furthermore, in the introduction of the current paper, the authors focus on the current lack of "treatments for cognitive impairment in HD. However, cognitive effects were not examined specifically, but instead combined with psychomotor effects to make a composite score. While the dangers of multiple comparisons must be considered, this composite method may actually be too conservative to show real effects, especially given (a) the small sample size, and (b) the fact that it combines the results of tasks that measure such different things; e.g. the Stroop word task versus finger tapping tasks. For example, the measure that showed the most improvement (across all patients) in the authors' 2018 paper (where activity NFT was used) appears to be the Stroop word task. In the current paper (for participants in the activity NFB condition) there again appears to be an (what looks like a significant) improvement in the Stroop work task. However, because the results of this task were combined with those of 6 other tasks, nothing of significance was found. I wonder if, had a larger sample size or a measure other than the composite score been used, the improvement in Stroop word task performance would have been found to correlate with NFT performance and/or near transfer? The authors conclude that "It is therefore doubtful whether NFT, even if it was successful, could lead to improved cognitive and psychomotor function in HD". However, I would say that based on the results (especially when the Stroop results are considered), this may again be a null finding simply due to low statistical power and due to the (composite) nature of analysis. While it is good to be conservative in statistical analyses, if there is not enough statistical power in analyses then strong conclusions about null-effects should not be made about non-significant results. Instead more samples should be collected to better test the hypothesis with more power.

   We have toned down and clarified the relevant points in the discussion on p.17.

   We agree with the reviewer that the Stroop word task shows consistently a large improvement at the first follow-up session in both our previous and our current work in the SMA activity NFT group. However, it is important to point out that a) the connectivity NFT treatment group did not show a comparable effect, and b) negative emotion recognition showed the largest improvement in our previous work, but this finding did not replicate in our current work. The word reading task is therefore an interesting finding, however it is a post-hoc finding and would need to be verified in follow-up research.

   We have now included a discussion on the Stroop word test as a potential measure for future research on pp. 18 and have toned down and clarified the relevant parts in the discussion section.

   > "*It is possible that by using a composite score we obscured large effects in specific measures. In addition to the composite score, we therefore also reported change in the individual measures (supplementary table 3 and supplementary figure 5). The Stroop word reading task shows a large increase in the first follow-up (mean (SD) = 5.75 (4.7)) in the activity NFT treatment group. This is in agreement with the results reported in our previous work (Papoutsi et al., 2018), where we also show a large improvement in this measure. However, this is a post-hoc finding and would need to be verified in future research.*

Taken together our findings are in agreement with recent studies that showed differences between treatment and controls groups during NFT, but weak evidence for near or far transfer (Schabus et al., 2017)".

Minor points:

4. The authors acknowledge that "A limitation of our study is that we could not dissociate the use of connectivity NFT from the use of intermittent feedback" but they do not give any explanation (that I could find) as to why they decided to use continuous feedback for the activity NFT condition and intermittent feedback for the connectivity NFT condition. Furthermore, the number of rest blocks (6 x 30s for the activity NFT condition and 5 x 45s for the connectivity NFT condition), the length of rest blocks (30s for the activity NFT condition and 45s for the connectivity NFT condition), and even the length of the overall NFT sessions, differed between these conditions (6x30s + 5x18s + 5x30s = 7 mins for the activity NFT condition; and 5x45s + 5x30 +5x3 = 6.5mins for the connectivity NFT condition). No explanation for these differences in task design between the two conditions is provided, and these differences provide too many potential confounds for a proper examination of activity versus connectivity NFT (this would have been a major point if significant differences had been found).

   We apologise for the lack of clarity in why we chose different paradigms for the two methods. The reason why we selected continuous feedback for the activity NFT group, was because this paradigm showed promising results in our previous work and is a more common paradigm for activity NFT. Introducing intermittent feedback would have been risky as it is unclear whether comparable results can be achieved with this approach. We used intermittent feedback for the connectivity group because in order to calculate reliable correlation coefficients for the two ROI time-series we needed at least 20-30 timepoints. This is in agreement with other connectivity neurofeedback studies, Yamashita et al. 2017, Fukuda et al 2015, Zilverstand et al. 2014. We have now clarified this point in the methods section on pp. 7-8.

   "*During the upregulation blocks feedback was presented continuously in the form of a red bar, similar to our previous pilot study (Papoutsi et al. 2018) which showed promising results. …*

   In the connectivity NFT runs feedback was presented intermittently at the end of the upregulation blocks in the form of a red bar. We selected intermittent instead of continuous feedback for the connectivity NFT because it provided reliable estimates of the ROI correlation by integrating over 30s consistent with previous research (Zilverstand et al., 2014; Megumi et al., 2015; Yamashita et al., 2017)".

   We apologise for the lack of clarity regarding the differences in the design between the two types of NFT runs. The two runs were structured differently, because of the difference in when the feedback was presented. The activity NFT runs were structured as Rest (30s) - Response (15s) - Upregulation (30s) repeated 5 times and ending with a final rest block (30s) so that we don't cut off the HRF for the last upregulation block. The connectivity runs were structured as Rest (30s) – Upregulation (30s) – Feedback (3s) – Rest (15s) repeated 5 times. There was no need to add an extra rest period in this case, because the upregulation block was followed by the feedback and rest blocks (18s).

   It is important to highlight that the critical part, the number and length of the upregulation blocks and the interval between the upregulation blocks were the same in both type of runs. In the activity NFT group, the response and upregulation blocks lasted 48s in total. Similarly, in the connectivity group, the feedback and rest blocks were 48s in duration. This was the interval between upregulation blocks.

We realize this was not clear in the text and have now edited the methods section on pp. 7-8 to explain this in more detail.

5. I cannot find any information about what the “response” is in the “response blocks” of the activity NFT condition. It does say “The rest and response blocks were identical to those of the transfer runs described previously.” Is this referring to the 2018 paper? This is not clear and in fact, since supplementary figure 2C shows a question mark on screen during the response blocks, it appears as if the participants need to make some behavioral response. The 2018 paper says “response blocks were 16 s to allow for the hemodynamic response to return to baseline prior to the upregulation blocks”. If the response blocks in the current paper function in the same way then this is understandable that this is necessary due to the continuous nature of the feedback in the activity NFT condition. However, why are response blocks 16s long in the 2018 paper and 18s in the current paper? And why there is no control for the presented stimulus (a question mark on the screen) in the connectivity NFT condition where feedback is intermittent? A better explanation of what the response blocks entail is needed.

We apologise for the lack of clarity due to the journal’s restrictions in word limit. The response blocks in the current paper function in exactly the same way as in our previous 2018 work. In our previous work we describe in detail the simple attention task that was used in the activity NFT condition. In more detail:

“To prevent participants from engaging in motor imagery during the baseline blocks and to provide an appropriate control condition, the baseline blocks consisted of a simple visual attention task that was closely matched to the neurofeedback training blocks. During the baseline blocks, the participants monitored changes in the luminance of the white bar, if the white bar flickered to grey (for 1s), they would wait until a question mark appeared inside the white bar and then make a response by clenching their left (non-dominant) fist once (supplementary figure 2). A maximum 3 out of 6 baseline blocks flickered per run and the timing of the flickering during the block was random”.

We have now updated the methods section on p. 7 to make it clearer.

> “*The rest and response blocks were identical to those of the near transfer runs and included a simple attention task as a control condition similar to our previous study (Papoutsi et al., 2018). During the rest blocks participants monitored changes in the luminance of the white bar, if the white bar flickered to grey (for 1s in 3 out 6 blocks), they would clench their left fist once, when the question mark appeared on screen (response block)*”.

The difference in the length of the response blocks between our current work and our previous work was due to differences in the EPI sequence used in the two different studies and specifically the TR. In our 2018 study the TR was 1s, whereas in this study the TR was 1.2s. Cogent was synchronized with the scanner volume acquisition and 18s was equivalent to 15 volumes.

With regards to the question why there is no control condition for the connectivity NFT. The feedback provided in the activity NFT group is percent signal change estimated using the mean of the rest period. The rest period is therefore very important for the activity NFT group and this is the reason why we include a simple attention task as a control condition. The rest period is not used for the calculation of the feedback in the connectivity NFT group, which uses the signal during upregulation from the 2 ROIs, hence there was no need to add an additional control condition.

6. On average, how many voxels were included in each ROI? What was the size of the rectangle drawn around each?

   We apologise for the omission. We have now added information on the average size of the ROIs on pp.8-9.

   > "*The mean (SD) number of voxels for the SMA ROI was 207 (82) and 156 (51) voxels for the activity and connectivity groups respectively. The mean (SD) size of the rectangles drawn was 8.5(1.7) x 10.3(3.1) x 9 and 7.7(1.4) x 8.8(1.7) x 9 voxels for the activity and connectivity groups respectively. There was a significant difference between the two groups in the size of the ROI (2-sample t-test t(30) = 2.12, p = 0.042). This was incidental, since the ROIs were drawn around significant clusters during the localizer.…*
   >
   > *The mean (SD) number of voxels for the striatal ROI was 92(83) and the mean (SD) size of the rectangle drawn was 4.9(0.7) x 7.1(1.5) x 9 voxels*".

   When compiling the information, we noticed that there was a significant difference in the size of the SMA ROI between the two groups. This was incidental, since the ROIs were drawn to encompass the activated cluster in SMA following the functional localizer, and possibly reflected within-subject variability in fMRI activation. Given the existing differences in the NFT signal between the two groups, i.e. percent signal change vs correlation coefficient, we do not believe that these differences are meaningful. In addition, both groups show an effect of training, there is therefore no reason to believe that the small difference in ROI size benefitted or disadvantaged any of the groups.

7. Why weren't the analyses adjusted for caudate volume atrophy like they were in the 2018 paper?

   Our 2018 study only included a treatment group, i.e. there was no control group and all analyses were therefore within-group. For this reason it was important to adjust for differences in age and disease stage between participants. We used caudate as our measure of disease stage. Our current study is an RCT and the groups have been randomized based on disease stage using the TMS. Using the TMS as a measure for randomization was more practical than using caudate volume, since the TMS score was available at screening.
