# Supplementary material for: Activity or connectivity? A randomized controlled feasibility study evaluating neurofeedback training in Huntington’s disease
Source: Brain Commun. 2020 Apr 23;2(1):fcaa049. doi: 10.1093/braincomms/fcaa049 (PMC7425518; doi:10.1093/braincomms/fcaa049)
Supplement: fcaa049_Supplementary_Data [file Revision_1_decision_letter.pdf › Revision_1_decision_letter.pdf]

BRAINCOM-2019-124

27-Mar-2020

Dear Dr. Papoutsi,

It is a pleasure to accept your manuscript entitled "Activity or Connectivity? A randomized, controlled feasibility study evaluating neurofeedback training in Huntington's disease" for publication in Brain Communications. Any comments made by the reviewer(s) are included below.

The next step is for your paper to go through "First Look". We will contact you again shortly if any final files or formatting changes are required for publication or if we have any queries.

If no changes are needed, your submission will be transferred to our Production Department and you will hear from the Production Editor in due course.

Thank you very much for your contribution. On behalf of the Editors of Brain Communications, we look forward to your future contributions to the Journal.

Yours sincerely,

Professor Kevin Talbot
Associate Editor
Brain Communications

Reviewer(s)' Comments to Author:

Reviewer: 1

Comments to the Author
The authors have amended the manuscript to my satisfaction.
